# “*Bifidobacterium longum*-reactive T helper cells as marker for intestinal barrier impairment in ICU patients with sepsis”

**DOI:** 10.1186/s13099-025-00770-9

**Published:** 2025-12-24

**Authors:** Lea-Maxie Haag, Markus Müller, Jörn Ziegler, Malte Lehmann, Julia Hecker, Rainer Glauben, Markus M Heimesaat, Friederike Compton, Britta Siegmund

**Affiliations:** 1 Department of Gastroenterology, Infectious Diseases and Rheumatology (including Nutrition Medicine, Charité – Universitätsmedizin Berlin, Universität Berlin and Humboldt-Universität zu Berlin, Berlin, Germany; 2 Berlin Institute of Health Charité, 10178 Berlin, Germany; 3 Gastrointestinal Microbiology Research Group, Institute of Microbiology, Infectious Diseases, and Microbiology, Charité – Universitätsmedizin Berlin, corporate member of Freie Universität Berlin and Humboldt-Universität zu, Berlin, Germany; 4 Department of Nephrology and Medical Intensive Care, Charité- Universitätsmedizin Berlin, Berlin, Germany; 5 Department of Gastroenterology, Infectious Diseases and Rheumatology (including Nutrition Medicine, Cluster of Excellence ImmunoPreCept, Charité – Universitätsmedizin Berlin, Universität Berlin and Humboldt-Universität zu Berlin, Hindenburgdamm 30, 12200 Berlin, Germany

**Keywords:** Intestinal barrier, Sepsis, Critical illness, Antigen-reacitve t cells, Intestinal dysbiosis, Intestinal barrier dysfunction

## Abstract

**Background:**

Critical illness often leads to the development of intestinal dysbiosis, which can have a significant impact on disease outcome. Intestinal barrier dysfunction is a common problem in intensive care unit patients, particularly those with sepsis. Despite its importance, early and reliable diagnosis of barrier dysfunction and evaluation of therapeutic options remain lacking in clinical practice. Given that intestinal hyperpermeability is associated with increased translocation of luminal antigens and subsequent priming of naïve T cells, we hypothesized that analysis of circulating peripheral antigen-reactive T cells could provide insight into the functionality of the intestinal barrier.

**Results:**

To test this hypothesis, 70 ICU patients were enrolled, including those with sepsis, those not meeting sepsis criteria, and COVID-19 patients, as well as 20 healthy volunteers. We identified a sepsis-specific T-helper cell signature in peripheral blood using the antigen-reactive T-cell enrichment (ARTE) technique followed by flow cytometric analysis. This signature was characterized by an expansion of gut trophic *Bifidobacterium longum*-reactive T-helper cells, indicating significant intestinal barrier dysfunction during sepsis.

**Conclusion:**

This approach allows the study of intestinal barrier functionality and provides a means to monitor the effects of potential therapeutic interventions over time using blood samples.

## Background

It is widely acknowledged that critical illness and intestinal function are inextricably linked. Patients who are critically ill and are being treated at an intensive care unit (ICU) may present with different gastrointestinal dysfunctions. Recent studies have highlighted the pivotal role of the microbiome in critical illness, particularly concerning disease progression and outcome [1]. Several factors in the ICU setting, including gastrointestinal dysmotility, antibiotic use, other drugs (non-steroidal anti-inflammatory drugs, proton pump inhibitors) altered diet, electrolyte imbalances, and systemic inflammation, promote the development of intestinal dysbiosis [2–5]. Dysbiosis is characterized by expansion of facultative pathogenic bacteria, such as *Proteobacteria*, a reduction in microbial diversity, and loss of potentially probiotic commensals (*Firmicutes* or *Bacteroidetes*), which are important for maintaining intestinal homeostasis. Prospective multicentre studies have confirmed the rapid and profound dysbiosis in critically ill patients and linked specific microbiota signatures to sepsis development and clinical outcomes [1, 6].

The functional consequences of dysbiosis include decreased production of short-chain fatty acids, reduced mucus production, and alterations in tight junction proteins, all of which contribute to epithelial barrier disruption and hyperpermeability [7–10]. Although intestinal barrier dysfunction in critically ill patients favors bacterial translocation and worsens disease severity, early and reliable diagnostic tools are lacking. Available tests are confounded by factors such as renal function and are logistically unfeasible in daily ICU care. Given the prognostic relevance of barrier integrity, this represents a significant gap in clinical management.

Given that intestinal hyperpermeability is associated with an increased translocation of luminal antigens, which in turn leads to the priming of naïve T cells and the potential for dysregulated immune responses toward commensal bacteria, we hypothesized that functional analysis of peripheral circulating commensal-reactive T cells could be used to characterize the functionality of the intestinal barrier. The lysates of *Escherichia* (*E.*) *coli*, *Candida albicans*, and *Bifidobacterium longum* were employed as representative commensal microbial antigens. Due to the low frequency and functional heterogeneity of antigen-reactive T cells in general, the detection and functional characterization of these cells presents a formidable challenge. However, these techniques may be of diagnostic and prognostic value [11–14]. This prompted us to employ antigen-reactive T cell enrichment (ARTE), a method based on CD154 detection and magnetic enrichment, which enables detailed phenotypical characterization of rare antigen-reactive T lymphocytes by multiparameter flow cytometry [11, 12, 14].

The aim of this study was therefore to investigate intestinal barrier function in critically ill ICU patients, specifically comparing those with sepsis, those without sepsis (referred to as “non-sepsis”), and patients with COVID-19, as well as healthy controls.

## Methods

### Study design, ethics and subjects

This prospective observational study was conducted at Charité – Universitätsmedizin Berlin, Germany. The study population consisted of ICU patients treated at the Department of Nephrology and Medical Intensive Care, Campus Benjamin Franklin. Blood samples were analyzed at the Department of Gastroenterology, Infectious Diseases and Rheumatology (including Clinical Nutrition), Campus Benjamin Franklin. All experiments were approved by the institutional review board of the Charité – Universitätsmedizin Berlin and conducted accordingly. Written informed consent was obtained from all patients and healthy volunteers. If a patient had a legal representative, consent was obtained from that representative. Eligible individuals were at least 18 years of age.

In total, 70 ICU patients were enrolled in the study, in addition to 20 healthy non-hospitalized volunteers who served as controls. The healthy controls had no history of any comorbidities listed in the exclusion criteria. ICU patients were allocated to one of three groups. Patients with a primary admission diagnosis of sepsis were classified according to the Sepsis-3 consensus definition, which defines sepsis as life-threatening organ dysfunction caused by a dysregulated host response to infection. Organ dysfunction was defined as an acute increase of at least two points in the Sequential Organ Failure Assessment (SOFA) score [15]. The diagnosis of sepsis in our study was established based on a combination of microbiological evidence, clinical examination, patient history, and imaging findings, supported by scoring systems including the SOFA and the Simplified Acute Physiology Score (SAPS II), as routinely applied in ICU practice. Septic shock was defined as sepsis with hypotension requiring vasopressor therapy in addition to lactic acidosis. The quick SOFA (qSOFA) score was additionally applied at admission to assist in rapid bedside screening and risk stratification.

Patients admitted to the ICU without evidence of sepsis but requiring intensive care for other reasons, such as neurological diseases (e.g., stroke or epilepsy), cardiovascular events (e.g., myocardial infarction), polytrauma, acute kidney failure, metabolic derangements, or intoxications, were included in the non-sepsis group. A further group comprised patients admitted to the ICU due to severe SARS-CoV-2 infection, confirmed by PCR testing. Although severe COVID-19 fulfils the Sepsis-3 definition of viral sepsis, we deliberately analyzed COVID-19 patients as a distinct subgroup and avoided the term viral sepsis. This decision was based on the fact that 9 out of 10 patients showed bacterial or fungal co-infections, making it impossible to attribute fulfilment of sepsis criteria solely to SARS-CoV-2 infection. Exclusion criteria were the presence of inflammatory bowel disease, celiac disease, short bowel syndrome, or a confirmed *Clostridioides difficile* infection. Patients with a stoma were also excluded.

### Definition and verification of infection

In all patients classified as septic, the diagnosis of infection was confirmed by positive microbiological culture, consistent with bacterial or fungal sepsis. Patients in the non-sepsis group showed no microbiological evidence of infection or were admitted to the ICU for non-infectious causes such as neurological or cardiovascular events. Patients with viral infections as the primary cause of sepsis were not included in the sepsis group. Instead, COVID-19 patients were analyzed separately as a distinct cohort. The higher inflammatory parameters and greater requirement for catecholamine therapy in the COVID-19 group reflect the hyperinflammatory phenotype and severe clinical course described in the literature for critically ill SARS-CoV-2 patients. It should also be noted that patient inclusion occurred on the day of ICU admission. At this early time point, all sepsis patients met the Sepsis-3 criteria of organ dysfunction (≥ 2 SOFA points), but not all had yet developed manifest organ failure requiring specific ICU interventions such as vasopressor or renal replacement therapy. This explains why the proportion of patients with overt organ failure at inclusion was below 100% while all fulfilled diagnostic criteria for sepsis.

### Blood sampling and routine blood analysis

In the course of routine blood sampling via an existing vascular access, peripheral blood samples (50 ml) were taken from for the purpose of isolating of peripheral blood mononuclear cells (PBMCs). The blood sampling was performed on the day of admission to the ICU. In addition to PBMC isolation for further analysis, routine laboratory blood tests performed as part of intensive care treatment relevant to the study included: C-reactive protein (CRP), leukocyte count, creatinine, urea, liver enzymes, bilirubine, procalcitonine, IgA, transglutaminase-antibodies and blood gas analysis. From healthy volunteers, blood was only drawn for PBMC analysis.

### PBMC isolation

PBMCs were isolated freshly from heparinized blood by density gradient centrifugation (Biocoll; Biochrom, Berlin, Germany). The separated PBMCs were washed once in phosphate-buffered saline (PBS) and once in RPMI 1640 (Gibco, Life Technologies, Darmstadt, Germany) supplemented with 1% penicillin/streptomycin (Merck, Darmstadt, Germany). Cells were counted using a Neubauer hemocytometer, adjusted to the desired cell density, and resuspended in RPMI1640 (Gibco, Life Technologies, Darmstadt, Germany) supplemented with 5% human AB serum (Sigma-Aldrich, St. Louis, MO, USA). Approximately 0.5–1.0 × 10^7^ PBMCs were then cultured overnight at 36 °C. Subsequently, PBMCs were stimulated with a range of commensal antigens, as well as Staphylococcus Enterotoxin B (SEB, serving as positive control), and a negative control. To identify rare antigen-reactive T cell subpopulations, the antigen-reactive T-cell enrichment (ARTE) technology was employed, and the cells were analyzed by flow cytometry.

### Bacterial lysates

Bacterial lysates were generated following previously described methods [16, 17]. In brief, *E. coli* ATCC25922 strain was cultivated on MacConkey agar plates (Oxoid, Wesel, Germany) for 24 h at 37 °C under aerobic conditions, whereas the *B. longum* ATCC20088 strain was cultured on Columbia agar supplemented with 5% sheep blood (Oxoid, Wesel, Germany) and incubated under anaerobic conditions (in a jar with an AnaeroGen gas pack; Oxoid, Wesel, Germany) for 48 h at 37 °C. The *C. albicans* SC5314 strain was cultivated on Sabouraud dextrose agar (Oxoid, Wesel, Germany) in an aerobic milieu (48 h, 37 °C). Following confirmation by light microscopy, the bacterial cells were harvested by scraping into 17.4 ml of distilled water and lysed by the addition of 0.4 ml of NaOH (1 M), 0.2 ml HCl (2 M), and 2 ml of 10X PBS (pH 7.5). The lysis of the cells was confirmed microscopically as well as by the negative culture of the lysates.

### Antigen-reactive T-cell enrichment

Antigen-reactive T cells were identified and enriched by applying the ARTE technique as previously described [18]. In brief, PBMCs were incubated for six hours with 1 µg/mL anti-CD40 (Miltenyi Biotec, Bergisch Gladbach, Germany) in the presence or absence of different antigens (*E. coli*,* B. longum*,* C. albicans*, SEB). After four hours, 1 µg/mL brefeldin A (Sigma Aldrich, Darmstadt, Germany) was added. Subsequently, cells were labeled with anti-CD154 (both cohorts) and anti-CD137 (only cohort 1) biotin antibodies followed by anti-biotin MicroBeads (CD154 & CD137 MicroBead Kit, Miltenyi Biotec, Bergisch Gladbach, Germany) and magnetically enriched with MS columns (Miltenyi Biotec, Bergisch Gladbach, Germany).

### Flow cytometric cell analysis

Surface staining was performed on the MS column. Samples were stained with either panel 1 (cohort 1) or panel 2 (cohort 2). *Panel 1* consisted of Brilliant Violet 510™ anti-human CD4; beriglobin, Pe/Cy7 anti-human CD8; PBS. *Panel 2* consisted of the following antibodies: Brilliant Violet 510™ anti-human CD4; Pe/Cy7 anti-ß1/CD29; beriglobin; PBS (all from BioLegend, Koblenz, Germany); VioBlue anti-α4/CD49; PE anti-ß7 (all from Miltenyi Biotec, Bergisch Gladbach, Germany). The negative fractions of MS columns were stored with a freezing medium at −80 °C. The enriched cell fractions were fixed with eBioscience™, FoxP3 staining buffer (Thermo Fisher Scientific, Waltham, MA USA), and subsequently intracellularly stained (*Panel 1*: APC anti-human IFNγ; APC/Cy7 anti-human IL-2; PerCP/Cy5.5 anti-human TNFα; all from BioLegend, Koblenz, Germany; FITC anti-human CD154 from Miltenyi Biotec, Bergisch Gladbach, Germany; *Panel 2*: PerCP/Cy5.5 anti-human IL-4; APC anti-human IL-10; APC/Cy7 anti-human IL-17a all from BioLegend; FITC anti-human CD154 from Miltenyi Biotec, Bergisch Gladbach, Germany). Flow cytometry analysis was performed on the FACS Canto II device (BD Bioscience, Heidelberg, Germany). Data were analysed with FlowJo analysis software (BD Biosciences, Ashland, OR, USA). Frequencies of CD4⁺CD154⁺ T cells were calculated as the proportion of CD4⁺CD154⁺ events within the total CD4⁺ T-cell population (CD4⁺ gate), in accordance with standard procedures for antigen-reactive T-cell enrichment (ARTE) analysis.

### Statistics

Statistical analysis was performed using GraphPad Prism software (GraphPad Software LLC, Version 8.0.2). In order to obtain an overview of the patient characteristics of the sepsis patients, using descriptive statistics mean and standard deviation of age, sex, organ failure, SAPS II, SOFA score, origin of sepsis, microbiology, catecholamine therapy, leukocytes, CRP, creatinine, and lactate were determined for the measurement. The test for statistical significance was performed using an unpaired t-test in the case of normal distribution, otherwise using the Mann-Whitney-U-Test. When comparing more than 2 groups, the ANOVA (normal distribution) or Kruskal-Wallace test was used. Since this study was a pilot study, a correction for multiple comparisons (Bonferroni correction) was omitted. Statistical significance was assumed at *p < 0.05, **p < 0.01, ***p < 0.001.

## Results

### Demographics and clinical characteristics

**Fig. 1:**
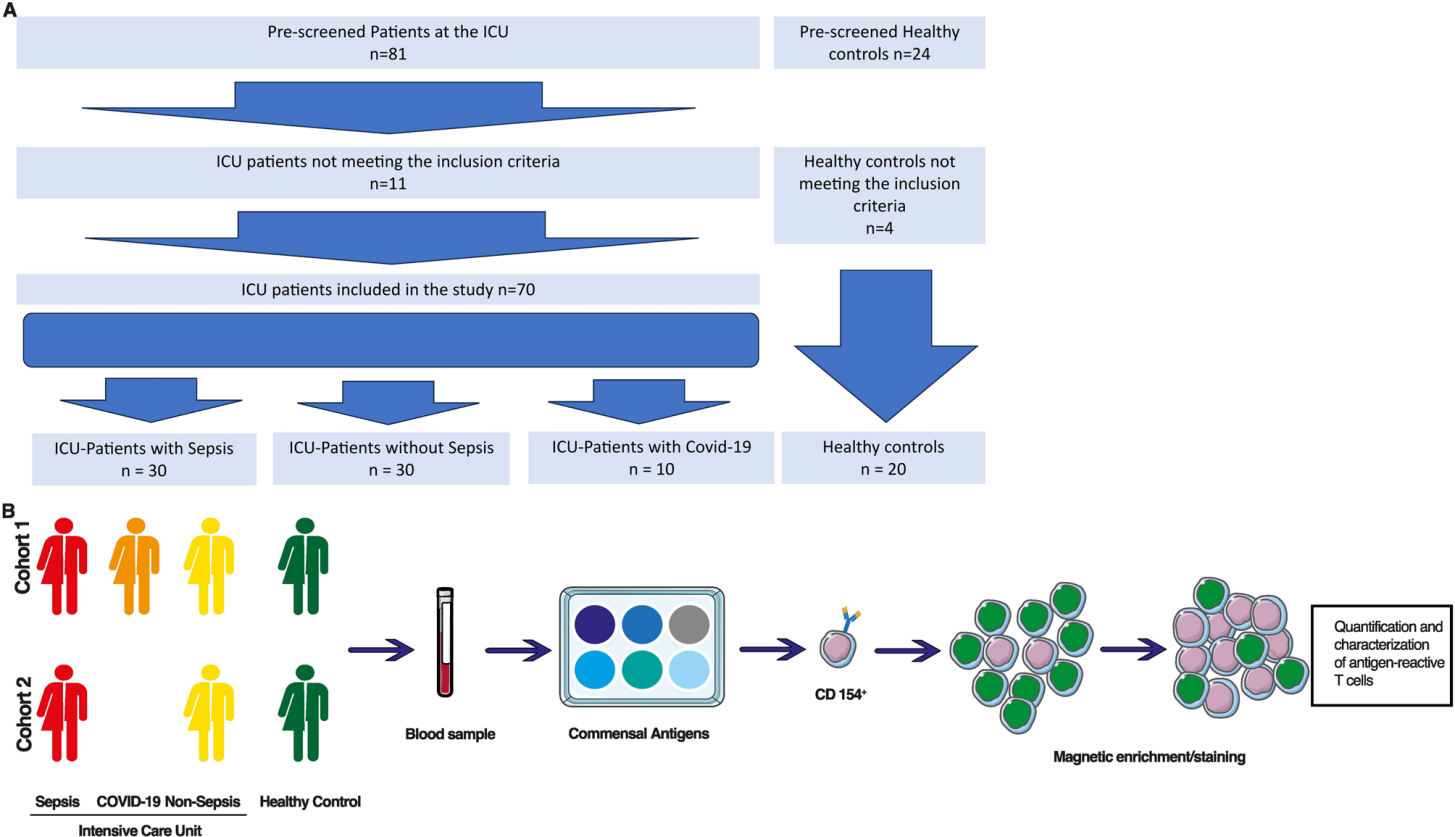
Flowchart of patient enrolment and illustration of the experimental setting (**A**) Flowchart of patient screening, enrollment, and allocation into study groups. A total of 81 ICU patients were pre-screened; 11 did not meet inclusion criteria. Seventy ICU patients were included in the study and assigned to the sepsis group (*n* = 30), non-sepsis group (*n* = 30), or COVID-19 group (*n* = 10). Twentyfour healthy controls were pre-screened, of which 4 did not meet inclusion criteria; 20 were included as healthy, non-hospitalized controls (**B**) Experimental workflow. Blood samples were collected at day 0 of ICU admission, stimulated with commensal microbial antigens, and analyzed using antigen-reactive T cell enrichment (ARTE) followed by multiparameter flow cytometry for quantification and characterization of antigen-reactive T-helper cells

A total of 70 ICU patients were included in this pilot study. Of these, 30 were diagnosed with sepsis, 30 did not meet the criteria for sepsis (referred to as “non-sepsis”) and 10 were diagnosed with COVID-19. In addition, blood was obtained from 20 healthy volunteers who were not hospitalized. The *first cohort* comprised 60 study participants, including 20 patients diagnosed with sepsis, 20 patients not meeting the sepsis criteria, 10 patients diagnosed with COVID-19, and 10 healthy volunteers, The *second cohort* consisted of an additional 30 participants, including 10 patients diagnosed with sepsis, 10 patients not meeting sepsis criteria, and 10 healthy volunteers. The analysis and characterization of systemic immune responses in both cohorts were focused on antigen-reactive T-cell subsets (Fig. 1). Table 1 provides a summary of the demographic data and clinical characteristics of cohort 1. The demographic data were matched among disease groups and healthy controls (Table 1). The median age of patients in the sepsis- and COVID-19-subgroups was 72 years (± 10 and ± 12 years, respectively), whereas the non-sepsis and healthy control subgroups had a median age of 71 years (± 11 and ± 9 years, respectively). In all subgroups, 60% of study participants were female. The proportion of female patients in the hospitalized subgroups (sepsis, non-sepsis, COVID-19) was 60% by coincidence in the context of these relatively small cohorts. For the healthy control group, we intentionally recruited 60% female volunteers in order to maintain a comparable sex distribution across groups. It should be noted that none of the patients in the sepsis or non-sepsis group received therapy with immunosuppressants including corticosteroids. In contrast, all patients from the COVID-19 subgroup were subjected to corticosteroid therapy. The SOFA scores were significantly higher in patients with sepsis and COVID-19 than in those without sepsis (6.7 ± 3.4 and 9.0 ± 2.4 vs. 1.5 ± 2.0; *p* < 0.001). With regard to the SOFA score, patients in the sepsis and COVID-19 subgroups exhibited significantly higher SAPS scores compared to non-sepsis ICU -patients (52 ± 15 and 53 ± 8.5 vs. 35 ± 11; *p* < 0.05). Neither the SOFA nor SAPS scores demonstrated any significant differences between the sepsis and the COVID-19 subgroups. Among the sepsis subgroup, the most prevalent infectious focus was the urogenital tract, followed by the lungs. In contrast, in the COVID-19 subgroup, no other disease focus than pulmonary was found.

The primary indications for admission to the ICU in the non-sepsis subgroup were cardiovascular and neurological disorders, including stroke and intracerebral hemorrhage. Approximately one-third of the non-sepsis patients presented with an infectious focus in the urinary or pulmonary systems. The rates of organ failure and the necessity for catecholamine therapy differed significantly between the groups. During intensive care therapy, 65% of patients in the sepsis subgroup and 100% of patients in the COVID-19 subgroup displayed organ failure, and catecholamine vasopressor therapy was carried out. All patients in the sepsis group fulfilled the Sepsis-3 criteria, i.e., exhibited organ dysfunction with an acute increase of at least two SOFA points, although not all had developed manifest organ failure requiring organ-supportive therapy at the time of inclusion. Conversely, 30% of patients in the non-sepsis subgroup met the criteria for organ failure (all patients with organ failure exhibited acute renal failure), necessitating the administration of catecholamine vasopressor therapy. The results of blood count examinations revealed significantly higher levels of leukocytes (16 ± 3/nl and 19 ± 16/nl vs. 9 ± 3/nl; *p* < 0.05 and *p* < 0.01) and CRP values (168 ± 99 mg/dl and 174 ± 123 mg/dl vs. 72 ± 112 mg/dl; *p* < 0.01 and *p* < 0.05) within the sepsis and Covid-19 subgroups compared to patients in the non-sepsis subgroup (Table 1). In the case of healthy control subjects, blood was only taken to isolate and further analyze PBMCs.

Lactate levels were found to be significantly elevated in the sepsis and COVID-19 subgroups when compared to the non-sepsis subgroup (22.50 mg/dl ± 22.44 and 18.80 ± 5.63 vs. 9.80 mg/dl ± 4.56; *p* < 0.005). Furthermore, creatinine levels were markedly elevated in the sepsis and COVID-19 subgroups compared to non-sepsis patients. Blood culture testing revealed a predominance of Gram-negative pathogens in both the sepsis and the COVID-19 subgroups, followed by Gram-positive cultures and positive fungal cultures (Table 1). For two patients in the sepsis subgroup, microbial diagnostics revealed growth in blood cultures without identification of a specific pathogen. Since microbiological examinations frequently revealed multiple pathogens per patient with positive blood cultures, Table 2 provides a summary of the distribution of all pathogens detected per subgroup. All patients classified as septic had microbiological confirmation of bacterial or fungal infection as the source of sepsis. Viral infections were not included as a primary focus in this group; patients with severe SARS-CoV-2 infection were analyzed separately as a distinct cohort. The Gram-negative bacterial species included *E. coli*, *Klebsiella pneumoniae*,* Klebsiella oxytoca*,* Pseudomonas aeruginosa*, and *Proteus mirabilis* (Table 2). Gram-positive bacterial species included *Staphylococcus aureus*, *Enterococcus faecalis*,* Streptococcus pneumoniae*, *Streptococcus agalactiae*, and *Streptococcus gorondij* (Table 2), whereas *Aspergillus* species and *Candida albicans* were detected in fungal cultures (Table 2). No positive cultures were found in the non-sepsis subgroup.

**Table 1:** Summary of demographic and clinical characteristics of cohort 1

	Sepsis	COVID-19	Non-sepsis	Healthy control
**n**	20	10	20	10
**Age** **(mean ± SD)**	72 ± 10	72 ± 12	71 ± 11	71 ± 9
**Female (%)** **Male (%)**	60 40	60 40	60 40	60 40
**SOFA Score** **(mean ± SD)** **(P-value)**	6.7 ± 3.4 < 0.001**	9.0 ± 2.4 < 0.001**	1.5 ± 2.0	-
**SAPS Score** **(mean ± SD)** **(P-value)**	52.0 ± 15.0 < 0.05*	53.0 ± 8.5 < 0.05*	35.0 ± 11.0	-
**Infectious origin of sepsis/focus of disease**				-
** Abdominal (%)**	5	0	0	-
** Pulmonary (%)**	40	100	20	-
** Urinary (%)**	55	0	10	-
** CNS (%)**	0	0	30	-
** Cardio-vascular (%)**	0	0	40	-
**Organ failure (%)**	65	100	30	-
**Catecholamine therapy (%)**	65	100	30	-
**Leukocytes/nl** **(mean ± SD)** **(P-value)**	16 ± 3 < 0.05*	19 ± 16 < 0.05*	9 ± 3	-
**CRP mg/l** **(mean ± SD)** **(P-value)**	168 ± 99 < 0.05*	174 ± 123 < 0.05*	72 ± 112	-
**Creatinine mg/dl** **(mean ± SD)** **(P-value)**	1.80 ± 0.94	2.31 ± 1.45	1.15 ± 1.14	-
**Lactate mg/dl** **(mean ± SD)** **(P-value)**	22.5 ± 22.44 < 0.05*	18.80 ± 5.63 < 0.05*	9.80 ± 4.56	-
**Microbiological data** **(main pathogen)**				-
** Gram-negative (%)**	50	40	0	-
** Gram-positive (%)**	25	20	0	-
** Fungi (%)**	15	30	0	-
** Positive cultures (%)**	10	0	0	-

Statistical significance as indicated *p<0.05, **p<0.01, ***p<0.001

CNS = central nervous system, CRP = C-reactive protein, SAPS = Simplified Acute Physiology Score, SD = standard deviation, SOFA = Sequential Organ Failure Assessment. All patients in the sepsis group fulfilled the Sepsis-3 criteria, which require organ dysfunction (≥2 SOFA points). The percentages listed under “Organ failure” indicate patients with manifest organ failure requiring specific ICU interventions (e.g., vasopressor support, renal replacement therapy) at the time of inclusion

**Table 2:** Summary and distribution of all pathogens in the Sepsis- and COVID-19-subgroup in cohort 1

Gram-positive bacteria	Gram-negative bacteria	Fungi
*Streptococcus pneumoniae* Sepsis: *n* = 3 COVID-19: *n* = 2	*E. coli* Sepsis: *n* = 5 COVID-19: *n* = 1	*Aspergillus fumigatus* Sepsis: *n* = 1 COVID-19: *n* = 3
*Enterococcus faecalis* Sepsis: *n* = 2 COVID-19: *n* = 1	*Klebsiella pneumoniae* Sepsis: *n* = 3 COVID-19: *n* = 3	*Candida albicans* Sepsis: *n* = 2 COVID-19: *n* = 1
*Staphylococcus aureus* Sepsis: *n* = 2 COVID-19: *n* = 1	*Pseudomonas aeruginosa* Sepsis: *n* = 3 COVID-19: *n* = 2	
Methicillin-resistant *Staphylococcus aureus* Sepsis: *n* = 1 COVID-19: *n* = 0	*Klebsiella oxytoca* Sepsis: *n* = 1 COVID-19: *n* = 0	
*Streptococcus agalactiae* Sepsis: *n* = 1 COVID-19: *n* = 0	*Proteus mirabilis* Sepsis: *n* = 1 COVID-19: *n* = 0	
*Streptococcus gorondii* Sepsis: *n* = 1 COVID-19: *n* = 0		

Table 1 only shows the main pathogens

The second cohort comprised 10 patients with sepsis and 10 patients without sepsis, all of whom were treated at the ICU. Additionally, 10 non-hospitalized healthy volunteers were included in cohort 2. Table 3 displays the main patient characteristics and demographic data of the second cohort, which were similar between the respective subgroups. As in cohort 1, there were significant differences in SOFA- and SAPS- scores between sepsis- and non-sepsis subgroups (7.6 ± 3.6 vs. 0.7 ± 0.7; *p* < 0.001 and 51.0 ± 17.0 vs. 26.0 ± 7.5; *p* < 0.001). Furthermore, the most prevalent source of sepsis were pulmonary and urinary infections, followed by abdominal infections (50, 40 and 10%, respectively). In the non-sepsis subgroup, the primary foci of disease were the central nervous and cardiovascular systems. The significantly higher incidence of organ failure and the need for catecholamine therapy in the sepsis subgroup observed in cohort 1 were confirmed in cohort 2. While there was no significant difference in leukocyte counts between sepsis and non-sepsis patients (10 ± 5 vs. 8 ± 2; *n.s.*), CRP (189 ± 123 vs. 42 ± 60; *p* < 0.05), lactate (26.1 ± 21.14 vs. 5.8 ± 1.99; *p* < 0.001) and creatinine (2.85 ± 2.15 vs. 1.03 ± 0.61; *p* < 0.05) values were significantly higher in the sepsis subgroup compared to the non-sepsis subgroup. Microbiological examinations revealed an equal proportion of Gram-negative and Gram-positive pathogens of 40% in the sepsis group as main pathogens, followed by positive fungal cultures of 10% (Table 3). Table 4 displays the identified pathogenic species within cohort 2.

**Table 3:** Summary of demographic and clinical characteristics of cohort 2

	Sepsis	Non-sepsis	Healthy control
**n**	10	10	10
**Age** **(mean ± SD)**	72 ± 12	69 ± 15	69 ± 9
**Female (%)**	60	60	60
**SOFA Score** **(mean ± SD)** **(P-value)**	7.6 ± 3.6 < 0.001**	0.7 ± 0.7	-
**SAPS Score** **(mean ± SD)** **(P-value)**	51 ± 17 < 0.001**	26.0 ± 7.5	-
**Infetious origin of sepsis/focus of disease**			-
** Abdominal (%)**	10	0	-
** Pulmonary (%)**	50	10	-
** Urinary (%)**	40	0	-
** CNS (%)**	0	60	-
** Cardio-vascular (%)**	0	30	
**Organ failure (%)**	70	10	-
**Catecholamine therapy (%)**	60	0	-
**Leukocytes/nl** **(mean ± SD)** **(P-value)**	10 ± 5 n.s.	8 ± 2	-
**CRP mg/l** **(mean ± SD)** **(P-value)**	189 ± 123 < 0.05*	42 ± 60	-
**Creatinine mg/dl** **(mean ± SD)** **(P-value)**	2.85 ± 2.15 < 0.05*	1.03 ± 0.61	-
**Lactate mg/dl** **(mean ± SD)** **(P-value)**	26.1 ± 21.14 < 0.005**	5.8 ± 1.99	-
**Microbiological data** **(main pathogen)**			-
** Gram-negative (%)**	40	0	-
** Gram-positive (%)**	40	0	-
** Fungi (%)**	10	0	-
** Positive cultures (%)**	10	0	-

Statistical significance as indicated *p<0.05, **p<0.01, ***p<0.001

CNS = central nervous system, CRP = C-reactive protein, SAPS = Simplified Acute Physiology Score, SD = standard deviation, SOFA = Sequential Organ Failure Assessment. All patients in the sepsis group fulfilled the Sepsis-3 criteria, which require organ dysfunction (≥2 SOFA points). The percentages listed under “Organ failure” indicate patients with manifest organ failure requiring specific ICU interventions (e.g., vasopressor support, renal replacement therapy) at the time of inclusion

**Table 4:** Summary and distribution of all pathogens detected in the Sepsis-subgroup in cohort 2

Gram-positive	Gram-negative	Fungi
*Streptococcus pneumoniae* *n* = 2	*Escherichia coli* *n* = 3	*Aspergillus species* *n* = 1
*Enterococcus faecalis* *n* = 2	*Klebsiella pneumoniae* *n* = 2	*Candida albicans* *n* = 1
*Staphylococcus aureus* *n* = 2	*Pseudomonas aeruginosa* *n* = 1	

Table 3 only shows the main pathogens listed

### Antigen reactive Th cell subsets as proxy of intestinal barrier function

Within this study, we employed a strategy linking intestinal barrier disruption and T cell activation. Using magnetic preenrichment followed by multiparameter flow cytometry, we were able to identify and characterize antigen-reactive Th subsets in the peripheral blood of ICU patients and healthy controls (Suppl. Figure 1 A). The intestinal microbiota consists of a variety of commensal microorganisms, including *E. coli*, *B. longum*, and *C. albicans*. These organisms were employed as microbial antigens in the present study. Given that intestinal barrier dysfunction is associated with increased bacterial translocation and subsequent priming of T cells, we hypothesized that the frequencies and functional signatures of commensal reactive Th subsets in peripheral blood would serve as readily accessible markers of intestinal barrier function to ultimately distinguish between patients diagnosed with sepsis from those not meeting the sepsis criteria.

### Antigen-reactive Th cells are increased in peripheral blood of sepsis ICU patients

**Fig. 2:**
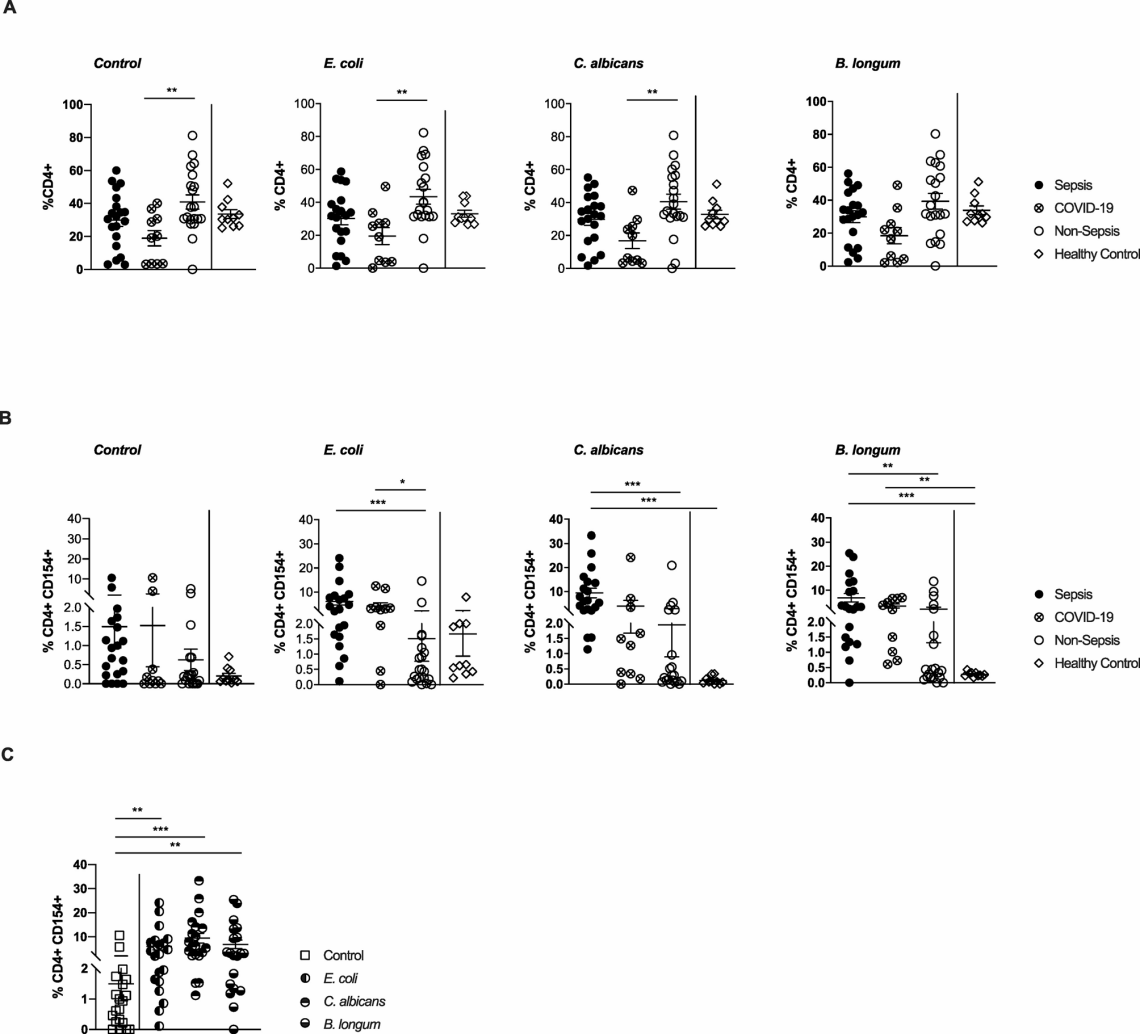
Frequencies of circulating CD4^+^ and CD4^+^CD154^+^ Th cells in cohort 1 (**A**) Frequencies of CD4^+^ Th cells after stimulation with control, *E. coli*,* C. albicans* or *B. longum* (*n* = 20/10/20/10). (**B**) Frequencies of CD4^+^CD154^+^ Th cells after stimulation with control, *E. coli*,* C. albicans* or *B. longum* (*n* = 20/10/20/10). (**C**) Frequencies of CD4^+^CD154^+^ Th cells in sepsis patients after stimulation with control or indicated antigens (*n* = 20). Data represented as mean ± SEM. *p < 0.05, **p < 0.01, and ***p < 0.001

Following stimulation in the presence (*E. coli*, *B. longum*, and *C. albicans)* or absence (control) of antigens, the overall frequency of CD4^+^ Th cells (calculated from the total number of CD4^+^ cells obtained after enrichment) did not differ significantly between sepsis- and non-sepsis patients. Among all patient subgroups, non-sepsis patients exhibited the highest median frequencies (Fig. 2A). Given that CD154 is expressed by all activated conventional CD4^+^ Th cell subsets and serves as marker of antigen-reactive T cells, antigen-reactivity was detected based on the upregulation of CD154 after ex vivo stimulation of PBMCs with respective antigens and subsequent magnetic enrichment (antigen-reactive T cell enrichment [ARTE]; Fig. 1; Suppl. Figure 1 A). Sepsis patients displayed significantly higher frequencies of *E. coli-*, *C. albicans-*, and *B. longum-*reactive CD154^+^ CD4^+^ Th cells in peripheral blood compared to patients not meeting the sepsis criteria (Fig. 2B). Furthermore, the frequencies observed in sepsis patients for *C. albicans-* and *B. longum-*reactive CD154^+^ Th cells were also significantly higher than in healthy control subjects (Fig. 2B). The level of *E. coli*-reactive CD154^+^ Th cells observed in patients with COVID-19 found to be intermediate between those observed in patients with sepsis and those observed in healthy controls. The levels of *B. longum*-reactive CD154^+^ Th cells observed in patients with COVID-19 were found to be significantly higher than those observed in healthy controls (Fig. 2B). Importantly, no statistically significant differences were detected between the respective cohorts following control stimulation (Fig. 2B). Focusing on the CD4⁺CD154⁺ T helper cell compartment, stimulation with commensal antigens led to an increase in activated cells in all groups; however, this effect was consistently more pronounced in septic ICU patients compared to non-septic ICU patients and healthy controls (Fig. 2C). These findings indicate that sepsis is associated with an enhanced antigen-specific activation of CD4⁺ T cells rather than a general increase in activation due to in vitro stimulation (Fig. 2C). Of note, the analysis of the regulatory T-cell compartment based on CD137 expression did not reveal significant differences in cell frequencies between groups (Suppl. Figure 2 A). As CD137 may also be transiently upregulated upon activation, these results should be interpreted with caution [18, 19]. Additional analysis including the regulatory marker FoxP3 in cohort 1 likewise showed no significant group-specific differences (not shown).

### Cytokine signatures in Circulating antigen-reactive Th cells

**Fig. 3:**
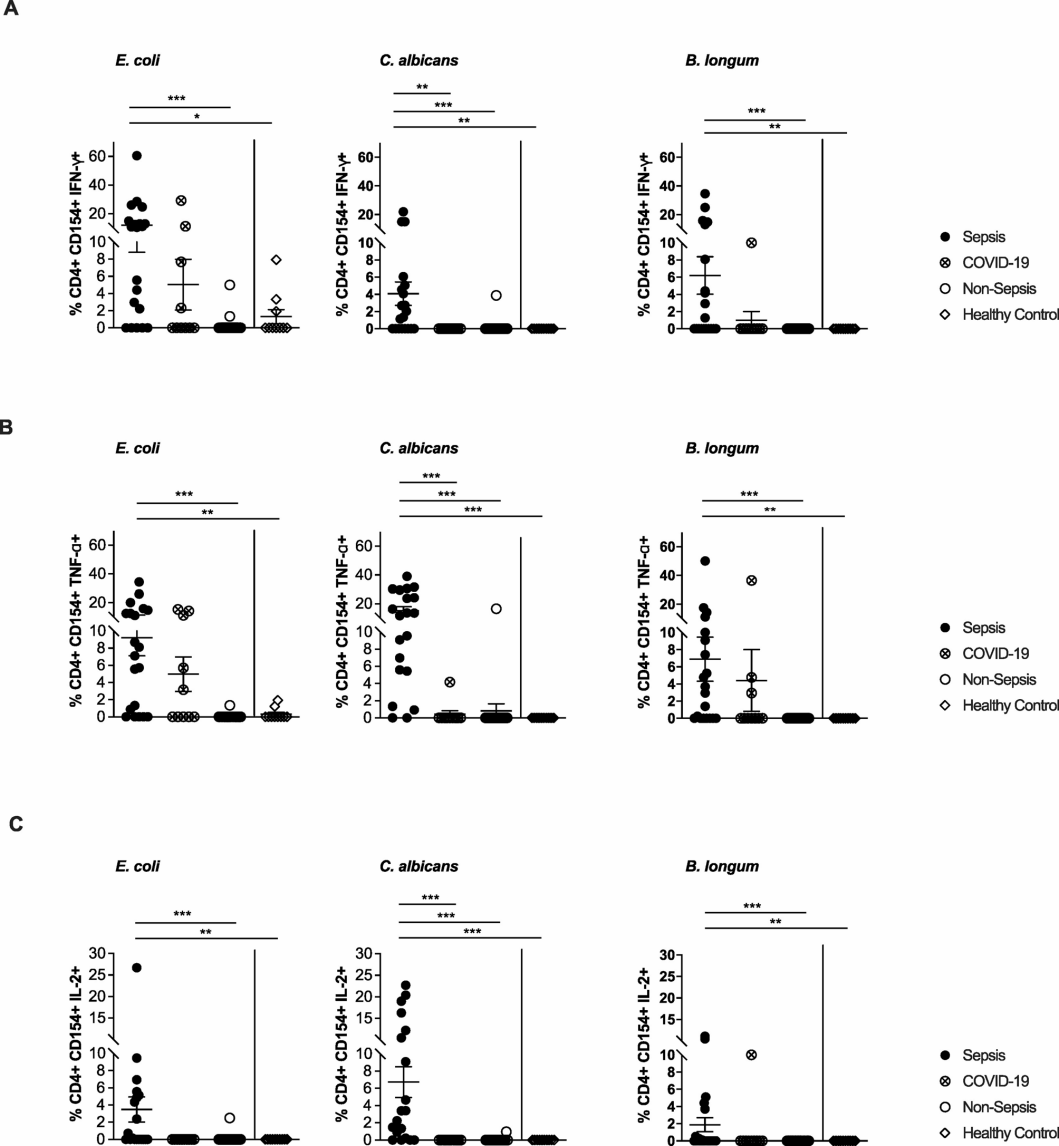
Cytokine production in antigen-reactive CD4^+^ CD154^+^ Th cells in cohort 1 (**A**) Frequencies of IFNγ producing CD4^+^CD154^+^ Th cells after stimulation with *E. coli*,* C. albicans* or *B. longum* (*n* = 20/10/20/10). (**B**) Frequencies of TNF-α producing CD4^+^CD154^+^ Th cells after stimulation with *E. coli*,* C. albicans* or *B. longum* (*n* = 20/10/20/10). (**C**) Frequencies of IL-2 producing CD4^+^CD154^+^ Th cells after stimulation with *E. coli*,* C. albicans* or *B. longum* (*n* = 20/10/20/10). Data represented as mean ± SEM. *p < 0.05, **p < 0.01, and ***p < 0.001

To further characterize the functionality of the antigen-reactive Th cell compartment, we focused on its pro-inflammatory capacity. Functional analysis of circulating *E. coli-*,* C. albicans- and B. longum-*reactive CD4^+^CD154^+^ Th cells revealed a significantly higher production of IFNγ and TNFα in sepsis patients compared to non-sepsis patients and healthy controls (Fig. 3A-B). The frequency of IFNγ- and TNFα-positive cells among *C. albicans* reactive CD4^+^CD154^+^ Th cells was also significantly higher in sepsis patients compared to the COVID-19 subgroup (Fig. 3A-B). Furthermore, CD4^+^CD154^+^ Th cells of sepsis patients exhibited a more pronounced IL-2 production upon stimulation with *E. coli*,* C. albicans*, and *B. longum* compared to non-sepsis patients and healthy controls (Fig. 3C). For *E. coli-* and *C. albicans-*specific Th cells, sepsis patients displayed higher IL-2 levels when compared to the COVID-19-subgroup (Fig. 3C).

The identification of the described peripheral antigen-reactive Th cell signatures that were distinct for sepsis patients led us to expand this study with a second cohort. In this cohort, we extended the functional characterizations by not only by including IL-17 as another pro-inflammatory cytokine but also by investigating the anti-inflammatory capacity of the antigen-reactive Th cell compartment. Furthermore, the analysis included the gut homing marker α4β7, to further investigate the relationship between the “sepsis-signature” and intestinal barrier function.

**Fig. 4:**
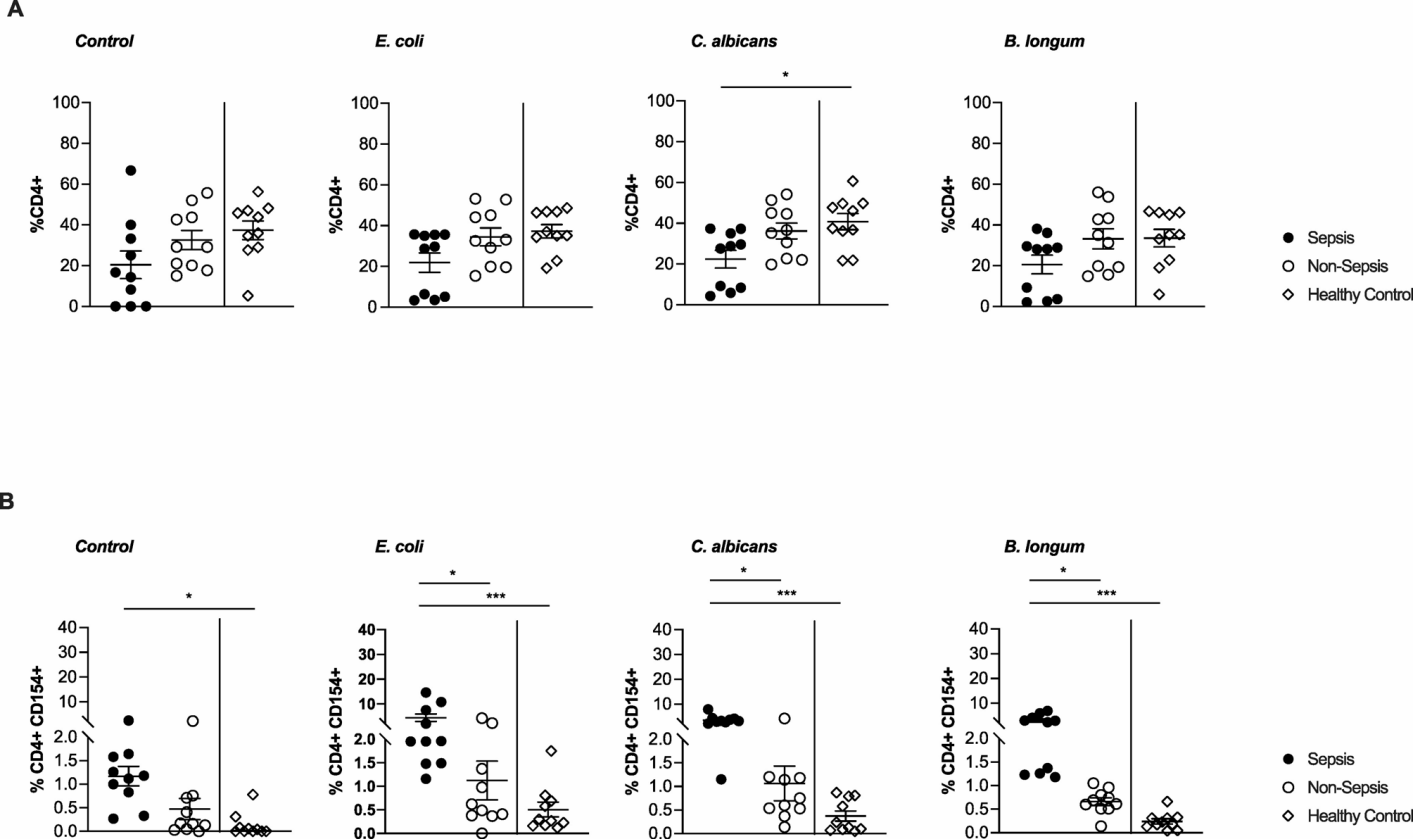
Frequencies of circulating CD4^+^ and CD4^+^CD154^+^ Th cells in cohort 2 (**A**) Frequencies of CD4^+^ Th cells after stimulation with control, *E. coli*,* C. albicans* or *B. longum* (*n* = 10/10/10). (**B**) Frequencies of CD4^+^CD154^+^ Th cells after stimulation with control, *E. coli*,* C. albicans*, or *B. longum* (*n* = 10/10/10). Data represented as mean ± SEM. *p < 0.05, **p < 0.01, and ***p < 0.001

**Fig. 5:**
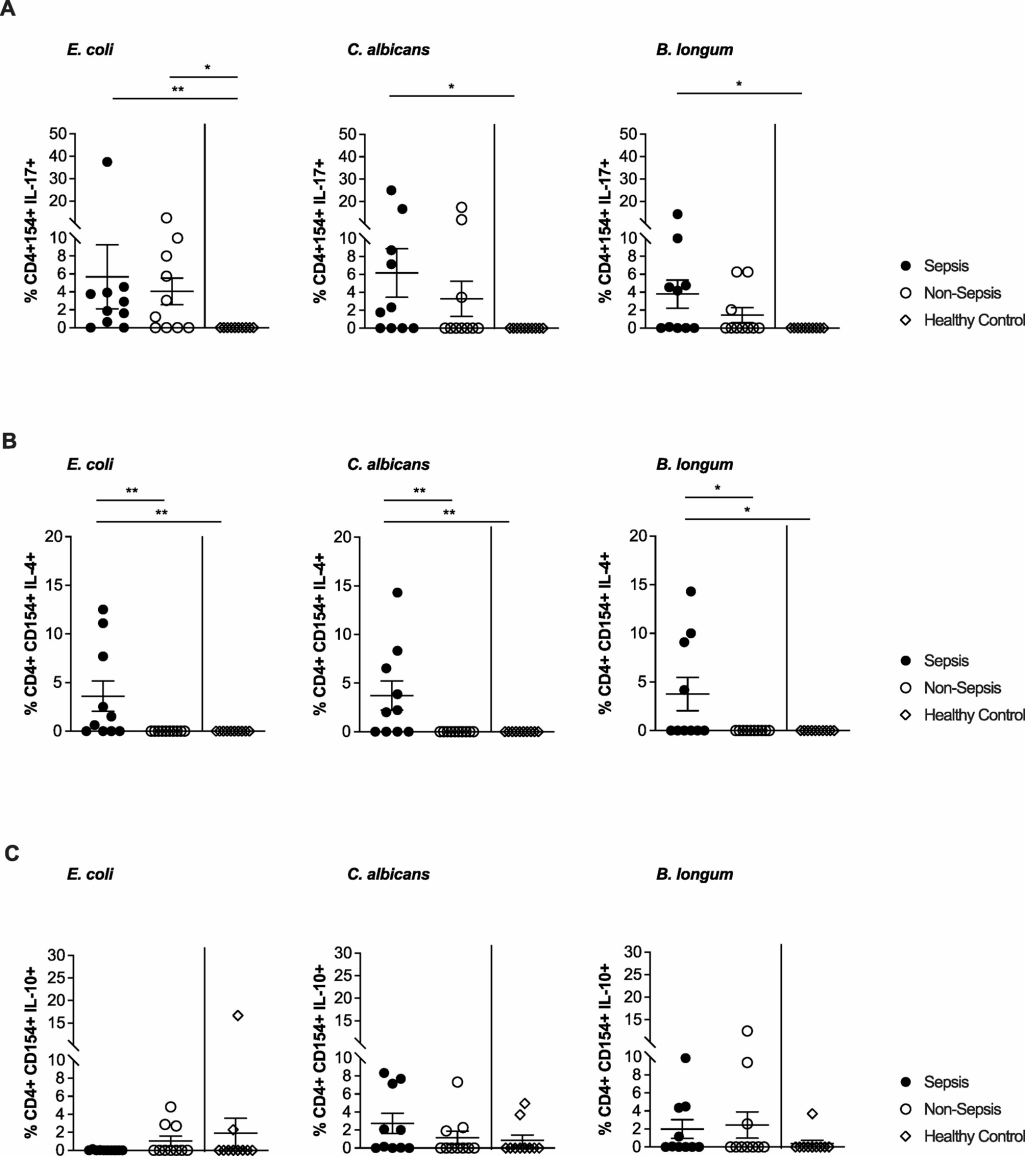
Cytokine production in antigen-reactive CD4^+^CD154^+^ Th cells in cohort 2. (**A**) Frequencies of IL-17 producing CD4^+^CD154^+^ Th cells after stimulation with *E. coli*,* C. albicans*, or *B. longum* (*n* = 10/10/10). (**B**) Frequencies of IL-4 producing CD4^+^CD154^+^ Th cells after stimulation with *E. coli*,* C. albicans*, or *B. longum* (*n* = 10/10/10). (**C**) Frequencies of IL-10 producing CD4^+^CD154^+^ Th cells after stimulation with *E. coli*,* C. albicans* or *B. longum* (*n* = 10/10/10). Data represented as mean ± SEM. *p < 0.05, **p < 0.01, and ***p < 0.001

As in cohort 1, the frequencies of total CD4^+^ Th cells did not differ significantly between sepsis and non-sepsis patients (Fig. 4A). Analysis in cohort 2 confirmed the distinct peripheral sepsis signature of antigen-reactive CD4^+^CD154^+^ Th cells. Indeed, also sepsis patients from cohort 2 showed significantly elevated frequencies of *E. coli-*,* C. albicans-* and *B. longum-*reactive Th cells in peripheral blood compared to critically ill patients not fulfilling the criteria for sepsis and healthy controls (Fig. 4B). Subsequent functional analysis of these antigen-reactive Th cells revealed that cells from sepsis patients exhibited the most pronounced production of IL-17 (Fig. 5A). When focusing on the anti-inflammatory profile of the antigen-reactive Th cell compartment, we observed a significantly higher IL-4 production in sepsis patients compared to non-sepsis and healthy counterparts (Fig. 5B). In contrast, the IL-10 levels were overall low and did not differ significantly between groups (Fig. 5C).

**Fig. 6:**
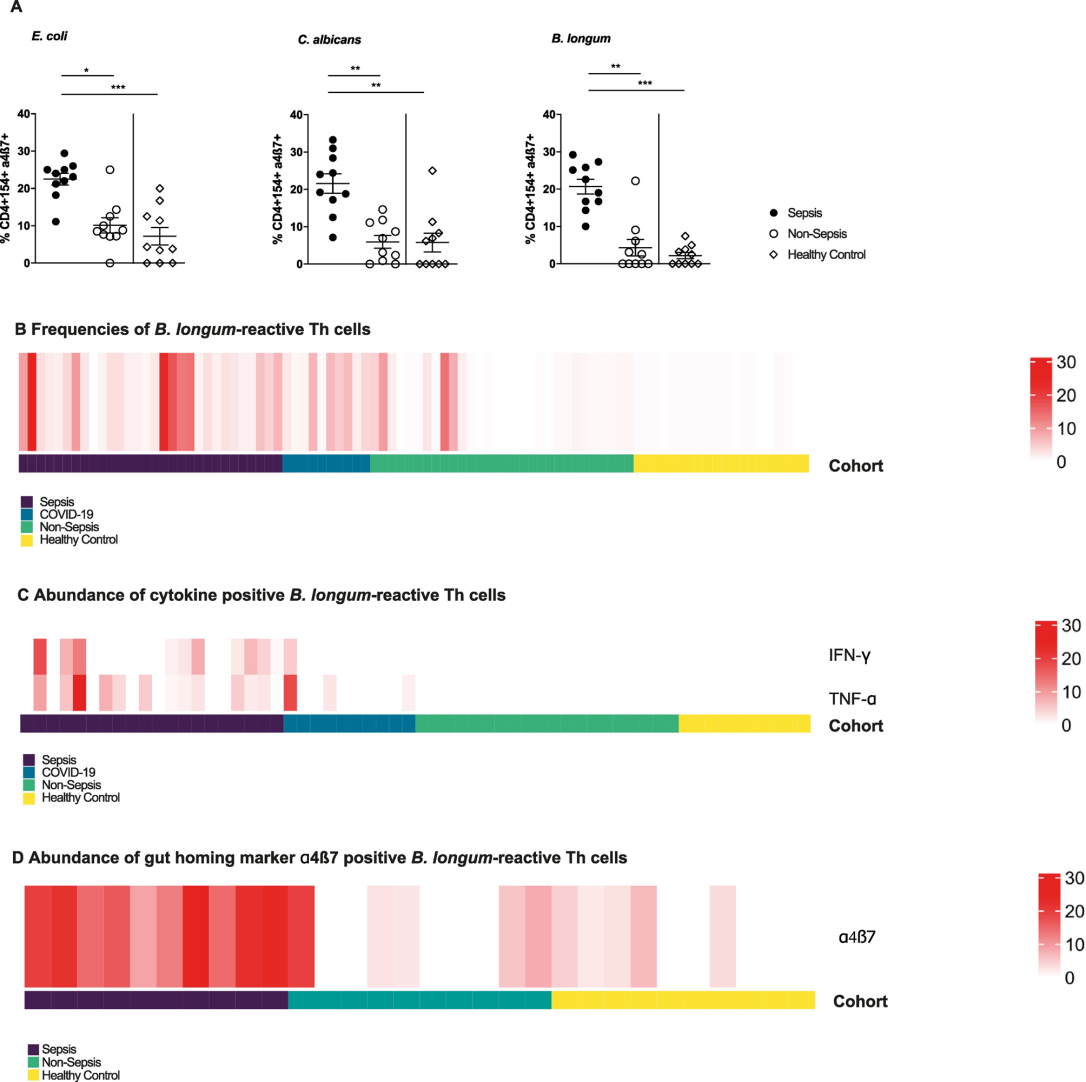
*Bifidobacterium longum*-reactive gut trophic Th cell signature (**A**) Frequencies of CD4^+^CD154^+^ α4ß7^+^ Th cells after stimulation with *E. coli*,* C. albicans*, or *B. longum* (*n* = 10/10/10). Data represented as mean ± SEM. *p < 0.05, **p < 0.01, and ***p < 0.001. (**B**) Frequencies of *B. longum* reactive Th cells in cohort 1 and 2. (**C**) Frequencies of IFNγ- and TNF-α producing *B. longum* reactive Th cells in cohort 1 and 2. (**D**) Frequencies of gut trophic α4ß7^+^ -expressing *B. longum-*reactive Th cells. (B) and (C) Merged data from both cohorts. Color code indicating respective groups

A comparison of the expression of the general gut homing marker α4β7 in sepsis patients, non-sepsis patients, and healthy controls revealed that sepsis patients had significantly higher frequencies of *E. coli-*,* C. albicans-*, and *B. longum-*reactive Th cells in peripheral blood positive for α4β7 than non-sepsis patients and healthy controls (Fig. 6A). A comprehensive view of a combined analysis of *B. longum-*reactive Th cells of both cohorts underscores the distinctive signature in sepsis patients, characterized by an expansion of proinflammatory, gut-trophic Th cells, indicative of a leaky intestinal barrier in sepsis ICU patients (Fig. 6B-D). To explore potential prognostic implications, we compared survivors (*n* = 20) and non-survivors (*n* = 10) within the sepsis subgroup. Non-survivors exhibited higher SOFA and SAPS-II scores as well as higher lactate levels at inclusion, whereas CRP and leukocyte counts were comparable between groups (Table 5). In addition, non-survivors showed a trend towards higher frequencies of *B. longum*–reactive CD4⁺CD154⁺ T cells and higher proportions of IFNγ- and TNFα-producing CD4⁺CD154⁺ T cells compared to survivors. Although not statistically significant, these data suggest a possible association between stronger commensal-reactive T-cell responses and adverse outcome in sepsis ICU patients.

**Table 5:** Subpopulation analysis: survivors vs. non-survivors in the sepsis cohort

	Non-survivors	Survivors
**Patients (n)**	10	20
**Ø SOFA Score**	8.2	6.4
**Ø SAPS-II Score**	58.4	49.2
**Ø CRP (mg/L)**	149.2	187.5
**Ø Leucozytes (/nL)**	13.9	13.7
**Ø Lactate (mg/dL)**	30.4	20.85

Statistical significance as indicated *p<0.05, **p<0.01, ***p<0.001

## Discussion

Intestinal barrier dysfunction is increasingly recognized as a decisive factor influencing outcomes in patients admitted to the ICU [20]. Despite its clinical importance, barrier dysfunction has so far been difficult to assess in ICU patients, as available diagnostic tests are logistically challenging and confounded by critical illness [21]. Our study provides novel functional evidence that circulating antigen-reactive T helper cells can serve as functional markers of intestinal barrier impairment in ICU patients, thereby linking immunological signatures with clinically relevant pathophysiology. The hypothesis that bacterial translocation of gut commensals could be a source and trigger of sepsis dates back several decades [22]. Intestinal barrier dysfunction has since been established as a key factor in the pathogenesis of sepsis, as increased permeability facilitates bacterial translocation, mucosal inflammation, and the maintenance of dysbiosis, resulting in a vicious cycle [23, 24].

Our data demonstrate a clear link between intestinal barrier disruption and systemic T-cell activation in ICU patients. The distinct “sepsis signature” detected in peripheral blood supports the concept that impaired barrier integrity contributes to the priming of commensal-reactive T cells. Moreover, elevated lactate levels observed in our sepsis subgroups are indicative of microcirculatory dysfunction, a well-recognized feature of sepsis pathophysiology, that likely exacerbates epithelial injury and promotes bacterial translocation [25, 26]. By analyzing two independent ICU cohorts - including patients with bacterial sepsis, non-septic critical illness, and COVID-19, as well as healthy controls, we were able to characterize circulating commensal-reactive T helper cells in unprecedented detail.

A particularly noteworthy finding was the expansion of *B. longum*-reactive CD4⁺ T helper cells in ICU patients with sepsis. This expansion represented the distinct “sepsis signature” identified in our study, characterized by the presence of pro-inflammatory, gut-trophic *B. longum*–reactive T cells in peripheral blood. *B. longum* belongs to the genus Bifidobacterium, one of the earliest colonizers of the human gastrointestinal tract and widely regarded as a probiotic organism with barrier-protective and immunomodulatory properties [27, 28]. Importantly, *B. longum* is considered non-translocating under physiological conditions and was not detected in blood cultures of our patients. The detection of systemic *B. longum*-reactive T cells in septic ICU patients therefore strongly suggests translocation across a compromised intestinal barrier rather than primary bloodstream infection. This “sepsis signature” may thus serve as a sensitive surrogate marker of intestinal barrier dysfunction in the ICU setting, complementing current diagnostic limitations and providing a functional immunological readout of barrier integrity [29]. The functional characterization of these antigen-reactive T cells further supports the existence of a distinct sepsis signature in ICU patients. Upon stimulation with commensal antigens, CD4⁺CD154⁺ T cells from septic ICU patients exhibited a markedly increased production of IFN-γ, TNF-α, IL-2, and IL-17 compared to non-septic ICU patients and healthy controls. This pro-inflammatory cytokine profile underscores the systemic immune activation associated with barrier dysfunction and is in agreement with earlier work linking disturbed intestinal barrier integrity to systemic inflammatory activation in critically ill ICU patients [30–33]. In contrast, anti-inflammatory cytokines such as IL-10 were generally low across all groups, and only a modest increase in IL-4 production was observed in ICU sepsis patients, which may represent a compensatory mechanism. These findings are consistent with previous reports showing complex patterns of anti-inflammatory cytokine responses, particularly for IL-10 and IL-4, in patients with sepsis and critical illness [34, 35]. Taken together, these data indicate that ICU patients with sepsis display a skewed pro-inflammatory immune response to commensal antigens, consistent with the notion that impaired barrier integrity drives systemic T-cell priming and contributes to the pathophysiology of sepsis. Further support for an intestinal origin of the circulating antigen-reactive T cells comes from our integrin expression analysis. Integrins are adhesion molecules essential for immune cell trafficking and tissue-specific homing [36, 37]. In humans, the expression of the integrin α4β7 on T cells identifies them as gut-trophic lymphocytes that preferentially migrate to intestinal and mucosal tissues. Our data showed that *B. longum*–reactive CD4⁺ T cells in septic ICU patients exhibited significantly higher α4β7 expression compared to non-septic ICU patients and healthy controls, indicating a gut-trophic phenotype. This finding strongly suggests that these antigen-reactive T cells were primed in the intestinal mucosa and subsequently entered systemic circulation as a result of barrier disruption. Taken together with the expansion of *B. longum*-reactive and pro-inflammatory T cells, the integrin data provide compelling evidence that the “sepsis signature” detected in our study originates from the gut and represents a functional correlate of intestinal barrier impairment in ICU patients with sepsis.

In addition to bacterial sepsis, we analyzed ICU patients admitted with severe COVID-19 infection to explore potential differences in immune activation. Although COVID-19 can meet the Sepsis-3 definition of viral sepsis, the immunological profile of these patients differed markedly from that of ICU patients with bacterial sepsis [38]. All COVID-19 patients in our study received systemic immunosuppressive therapy at the time of ICU admission, which likely blunted pro-inflammatory T-cell responses and may explain the absence of a comparable cytokine signature. Furthermore, 9 of 10 COVID-19 patients had positive bacterial or fungal cultures during their ICU stay, complicating attribution of the observed immune responses solely to SARS-CoV-2 infection. These findings emphasize that immune activation in COVID-19 ICU patients is strongly shaped by both the therapeutic immunosuppression and the high rate of secondary infections, and that the characteristic *B. longum*-reactive T-cell expansion observed in bacterial sepsis represents a unique feature of this condition rather than a general phenomenon of critical illness. To further explore the clinical implications of our findings, we performed a subpopulation analysis within the sepsis cohort comparing survivors and non-survivors during the ICU stay. Although limited by sample size, non-survivors displayed higher SOFA and SAPS-II scores as well as elevated lactate levels, consistent with more severe organ dysfunction. Importantly, non-survivors also showed a trend toward higher frequencies of *B. longum*-reactive CD4⁺CD154⁺ T cells and greater production of IFN-γ and TNF-α compared with survivors. These results suggest that dysregulated commensal-reactive T-cell responses may accompany, or even amplify, disease severity in sepsis. While these data do not establish causality, they support the concept that systemic immune activation resulting from intestinal barrier breakdown could have prognostic relevance in ICU patients [20]. Larger prospective studies will be required to validate these trends and to determine whether commensal-reactive T-cell signatures might serve as biomarkers for clinical outcomes in sepsis in the intensive care setting. While no direct permeability assays, such as the lactulose/mannitol absorption test, were performed in this pilot study, several factors strengthen the interpretation that the observed immune signatures reflect intestinal barrier dysfunction. None of the septic ICU patients showed microbiological evidence of *B. longum* infection, yet they displayed pronounced *B. longum*-reactive and gut-trophic T-cell responses, consistent with translocation of commensal antigens across a compromised barrier. Integrin α4β7 expression analyses further support a gut origin of these cells. Nevertheless, combining the described immunological approach with established permeability assays in future studies would be highly valuable to validate and extend these findings.

In summary, our study identifies a distinct immunological “sepsis signature” in ICU patients, characterized by the expansion of pro-inflammatory, gut-trophic *B. longum* -reactive CD4⁺ T helper cells. The accompanying cytokine and integrin expression profiles indicate that these cells are likely primed in the intestinal mucosa and reflect disruption of the epithelial barrier. This blood-based immune signature provides a novel approach to functionally assess intestinal barrier integrity in critically ill patients, a parameter that has so far remained difficult to evaluate with existing diagnostic tools. Beyond its mechanistic insights, the detection of commensal-reactive T cells may have prognostic and therapeutic relevance, potentially serving as a biomarker to monitor barrier function and to evaluate interventions aimed at restoring intestinal homeostasis in the ICU setting. Future studies in larger and longitudinal patient cohorts are warranted to validate these findings and to determine whether modulation of intestinal barrier integrity or targeted immune regulation could improve outcomes in sepsis and other critical illnesses.

## Data Availability

The data supporting this study are securely stored on the Charité – Universitätsmedizin Berlin server. Access may be granted upon reasonable request to the corresponding author or relevant university department, subject to institutional policies and ethical considerations.

## Abbreviations

ICU: Intensive care unit
SCFA: Short–chain fatty acids
ARTE: Antigen–reactive T cell enrichment
Th: T helper
SOFA: Sequential organ failure assessment
PBMCs: Peripheral blood ononuclear cells
CRP: C–reactive protein
PBS: Phosphate–buffered saline
CNS: Central nervous system
SAPS: Simplified acute physiology score
SD: Standard deviation
IL: Interleukin
TNF: Tumor necrosis factor
IFN: Interferon
